# TP73-AS1 promotes gastric cancer proliferation and invasion by regulation miR-27b-3p/TMED5 axis

**DOI:** 10.7150/jca.66683

**Published:** 2022-02-07

**Authors:** Chenhui Bao, Lin Guo

**Affiliations:** Department of General surgery, ShengJing Hospital of China Medical University, No. 36 Sanhao Street, Heping District, Shenyang City, Liaoning Province, China 110004.

**Keywords:** Gastric cancer, TP73-AS1, miR-27b-3p, TMED5, proliferation, invasion, wnt/β-catenin

## Abstract

**Background:** Gastric cancer (GC) is a common gastrointestinal malignancy. Evidence suggests that long non-coding RNAs (lncRNAs) influence mRNA expression to induce GC progression. We aim to investigate the function and regulatory mechanism of TP73-AS1 in GC.

**Materials and methods:** We detected TP73-AS1, miR-27b-3p, and TMED5 (Transmembrane P24 Trafficking Protein 5) by real-time polymerase chain reaction (RT-PCR). Similarly, the protein levels of TMED5 and wnt/β-catenin were detected by western-blot. The colony formation and Cell-Counting Kit-8 (CCK-8) assay detected cell proliferation. Transwell and scrape assay tested cell migration and invasion. Dual-luciferase reporter assays confirmed directed binding targets. Tumor xenograft in nude mice checked the result *in vivo*.

**Results:** TP73-AS1 over-expressed in GC. Suppressed TP73-AS1 inhibited cell proliferation, migration, and invasion. However, down-regulated miR-27b-3p could reverse the effects of weakenTP73-AS1 on the progression of GC. Moreover, TMED5 was also up-regulated in GC. Both TP73-AS1 and TMED5 were the direct target of miR-27b-3p. Meanwhile, miR-27b-3p was a negative correlation with TP73-AS1 and TMED5. The TP73-AS1/miR-27b-3p/TMED5 axis regulate wnt/β-catenin pathway.

**Conclusion:** TP73-AS1 promoted GC proliferation, migration, and invasion by sponging miR-27b-3p to regulate TMED5. TP73-AS1 was a potential onco-lncRNA in GC.

## Introduction

Gastric cancer (GC) is a common malignancy worldwide, and it is associated with high mortality 1. In 2020, there were 9.96 million cancer-related deaths worldwide, including 770,000 cases of GC, making it the fourth most significant contributor to cancer-related death. Owing to population aging, it is expected that by 2040, the global cancer burden will be 50% higher than that in 2020 2, 3. Despite significant progress in surgery, radiotherapy, and chemotherapy for GC, the 5-year overall survival (OS) rate in cases of advanced GC remains lower than 30% owing to a lack of sensitive and specific biomarkers. Therefore, identifying new sensitive biomarkers is critical for prognostication and individualized treatment in GC 4.

Non-coding RNAs (ncRNAs) have been the essential players in the cancer-against arena for the past decades. Examining the function of ncRNAs in normal and diseased tissues and elucidating their relationship to cancer remains a challenge. We still need to explain whether ncRNA is the missing piece in the cancer jigsaw puzzle. Our previous study found that miR-27b-3p was weakly expressed in GC, and it could be involved in the malignant phenotype by regulating the Hippo pathway 5. In addition, miR-27b was also reported to inhibit the metastasis and proliferation of GC cells by acting on NR2F2 and ROR1, respectively 6, 7. Therefore, it is vital for us to analyze the function of miR-27b-3p in GC further.

In addition to miRNAs, ncRNAs include long-stranded ncRNAs (lncRNAs), circular RNAs (circRNAs), and others. Among them, lncRNAs have made considerable progress in cancer-related research. Long non-coding RNA (lncRNA) is a class of 200-100000 nucleotides transcripts lacking protein-encoding functions 8. GC-related lncRNAs research had led to valuable discoveries in several fields. LncRNA MACC1-AS1 maintained stemness and promoted chemoresistance in GC by regulating fatty acid oxidative metabolism 9. Circulating exosomal lncRNAGC1 as a molecular marker for early detection and monitoring of GC progression 10. LncRNA PVT1 induced invasive angiogenesis through activation of STAT3/Slug axis 11. Because lncRNAs are similar to message RNAs (mRNAs) in structure, they also have commonalities in regulation. It is well known that miRNAs can inhibit mRNA transcription and subsequent function by binding to the 3'UTR (3'-untranslated region) 12. Similarly, miRNAs can bind to the 3'UTR of lncRNAs, thereby affecting the transcription of lncRNAs. It inspires us that lncRNA can specifically bind to miRNAs to relieve miRNA's inhibition of mRNA. This mechanism is known as competitive endogenous RNAs (ceRNAs) 13. Therefore, the search for lncRNAs involved in regulating miR-27b-3p may be of great benefit for further analysis of their functions in GC.

In this study, we found that lncRNA TP73-AS1 (TP73-AS1) was overexpressed in GC. Mechanically, TP73-AS1 could competitively bind miR-27b-3p, thereby promoted TMED5 (Transmembrane P24 Trafficking Protein 5) expression. TP73-AS1/miR-27b-3p/TMED5 axis activated wnt/β-catenin pathway, assisting GC invasive and migration.

## Materials and methods

### Cell culture

The human normal gastric epithelial cell line (GES-1) and human GC cell lines (AGS, HGC27, MUGC3, and MKN45) were acquired through the central laboratory of Shengjing Hospital. Cells were cultured in RPMI-1640 medium (Gibco, USA) with 10% fetal bovine serum (FBS, Gibco, US) as previously described 5.

### Tissue samples

A total of 40 paired GCs and adjacent normal tissues were surgically obtained from Shengjing Hospital of China Medical University. The specimens were stored at -80 °C. Each participant had a clear pathological diagnosis and was staged based on AJCC (8^th^) TNM criteria. Enrolled patients had not received any preoperative treatment and had no other tumors or systemic diseases that shortened survival. The information of the patients were shown in Table 1. The study was reviewed and approved by the Faculty of Science Ethics Committee at ShengJing Affiliated Hospital of China Medical University (2016PS326K). All patients signed a written informed consent before surgery.

### RNA isolation and RT-PCR

The total RNA of samples was separated and extracted by TRIzol (Invitrogen, USA). cDNA was obtained by the RevertAidTM First Strand cDNA Synthesis Kit (Fermentas, Canada). PCR was running in an ABI SYBR Green Master Mix (Invitrogen, USA). The data were analyzed using the 2^-△△Ct^ method, as mentioned before 5. The primers sequences were as follows, TP73-AS1: 5'-CTCCGGACACTGTGTTTTCTC-3' and 5'- TCTTTTAAGGCGGCCATATC-3'; miR-27b-3p: 5'- TCTGGGCAACAAAAGTGAG-3' and 5'- CTCAACAGGTGTCGTGGA-3'; TMED5: 5'- CCTTTCTACCCTTGATTT -3' and 5'-TATAGCCACATTCTCCTT -3'; U6: 5'- CTCGCTTCGGCAGCACA-3' and 5'- AACGCTTCACGAATTTGCGT-3'; β-actin: 5'-ATTGGCAATGAGCGGTT-3' and 5'-CGTGGATGCCACAGGACT-3'.

### RNA fluorescence *in situ* hybridization

GC cells were fixed with 4% formaldehyde for 15 minutes, washed with PBS, treated with pepsin, and dehydrated with ethanol. According to the manufacturer's instructions, RNA fluorescence *in situ* hybridization (FISH) was performed using a RiboTM Fluorescent *In situ* Hybridization Kit (RiboBio, China). The 4',6-diamino-2-phenylindole (DAPI) was performed to stain DNA. The GFP-labeled TP73-AS1 probe was detected and observed by Leica-SP8 confocal microscope (Leica, Germany).

### Western blot

RIPA lysis buffer (Beyotime, China) and BCA protein assay kit (Beyotime, China) were used to extract and measure protein, respectively. The details of the process were described in our previous study 5. The following antibodies: anti-TMED5 antibody (1:1000, Abcam, USA) and anti-β-actin antibody (1:1000, Santa Cruz, USA), 2^nd^ antibody (1:2000, Abcam) were used.

### Nuclear and cytoplasmic separation assay

The PARIS kit (Thermo Fisher Scientific, Japan) divided the total cellular fractions into nuclear and cytoplasmic fractions. The kit separated nuclear and cytoplasmic components before RNA extraction made sure that the RNA was isolated from the same sample.

### Immunohistochemistry

GC tissue serial sections were fixed on poly-L-lysine-coated slides. The sections were incubated with anti-TMED5 (1:1000, Invitrogen, USA) and visualized by incubation with biotin-conjugated secondary antibodies and diaminobenzidine substrate (BOSTER, China). Immunohistochemistry was scored according to the intensity of staining and the proportion of positive cells, and they were blind review by two pathologists.

### CCK-8 assay

5×10^4^ cells/well were seeded in a 96-well plate and incubated at 37 °C for 24, 48, 72, or 96 hours. Each well was added with 10 μl of thawed CCK-8 solution (Beyotime, China). Subsequently, the cells were incubated for 2h. A micro-plate analyzer (Molecular Devices, USA) detected the absorbance at 450 nm.

### Colony formation assay

After transfection, 1×10^3^ cells were seeded into 6-well plates and incubated for 2 weeks. PBS washed the cell colonies three times. Moreover, cells were fixed with 4% paraformaldehyde and stained with 0.1% crystal violet solution (Sigma, USA). The number of stained colonies was observed using a light microscope.

### Transwell

Transwell chamber (8 μm pore size, Millipore, USA) coated with Matrigel (BD Biosciences, USA). 1 × 10^5^ cells/well were seeded into the upper chamber with serum-free culture medium. Then, a culture medium containing 20% FBS was added to the lower chamber. The cell stayed in the top chamber was carefully removed using a cotton swab after incubation for 24h at 37 °C. The cells on the lower chamber were fixed with 70% ethanol for 10 mins and stained with 0.1% crystal violet for 15 mins. The invasive cells were counted by ImageJ software (USA).

### Scrape

Cells were plated into a 6-well plate (Corning, USA). After 24 h incubation, a wound gap was created by a 200 μl pipet. Images of wound monolayers were acquired using All-in-One Fluorescence Microscope BZ-X800 (Keyence, Japan) at 0 and 48 h post-wounding.

### Luciferase and TOPFlash/FOPFlash reporter assay

We performed a dual-luciferase reporter assay to confirm whether miR-27b-3p could bind to the 3'UTR of TP73-AS1 and TMED5. The 3'-UTR wild and mutant type sequences of TP73-AS1 and TMED5 were cloned into the psiCHECK2 vector (Promega, USA). Cells were seeded in 24-well plates, growing to approximately 70% confluence, and co-transfected with luciferase plasmids and miR-27b-3p mimics or control using POLO3000 transfection reagent (Research and science, China). After 48h transfection, the luciferase activity was determined by Multiskan FC Microplate Reader (Thermo Fisher Scientific, USA).

Wnt/β-catenin signaling reporter TOPFlash/FOPFlash (Upstate Biotechnology, USA) was co-transfected into cells along with TP73-AS1 vectors, TMED5 vectors, or miR-27b-3p mimics. Experiments were performed in AGS.

### Tumor xenograft in nude mice

BALB/c nude mice (age five weeks, female, weight 20-22g, Beijing Vital River Laboratory Animal Co., Ltd, China) were housed in a specific pathogen-free environment. The cell suspension was then injected subcutaneously into the flanks of nude mice (0.1 ml, 1×10^7^ cells/ml). All nude mice were euthanized after 3 weeks. The tumors were collected and weighed. All animal experiments and operations were conducted according to the Animal Care and Use Committee of Shengjing Hospital of China medical university.

### Statistical analysis

All statistical analyses were performed using SPSS 23.0 software. The quantitative data derived from three independent experiments are expressed as mean ± standard deviation (SD). Significance was determined by one-way ANOVA to t-test. Values of *P*<0.05 were considered as statistical significance.

## Results

### TP73-AS1 is a target of miR-27b-3p in GC

**Figure 1:**
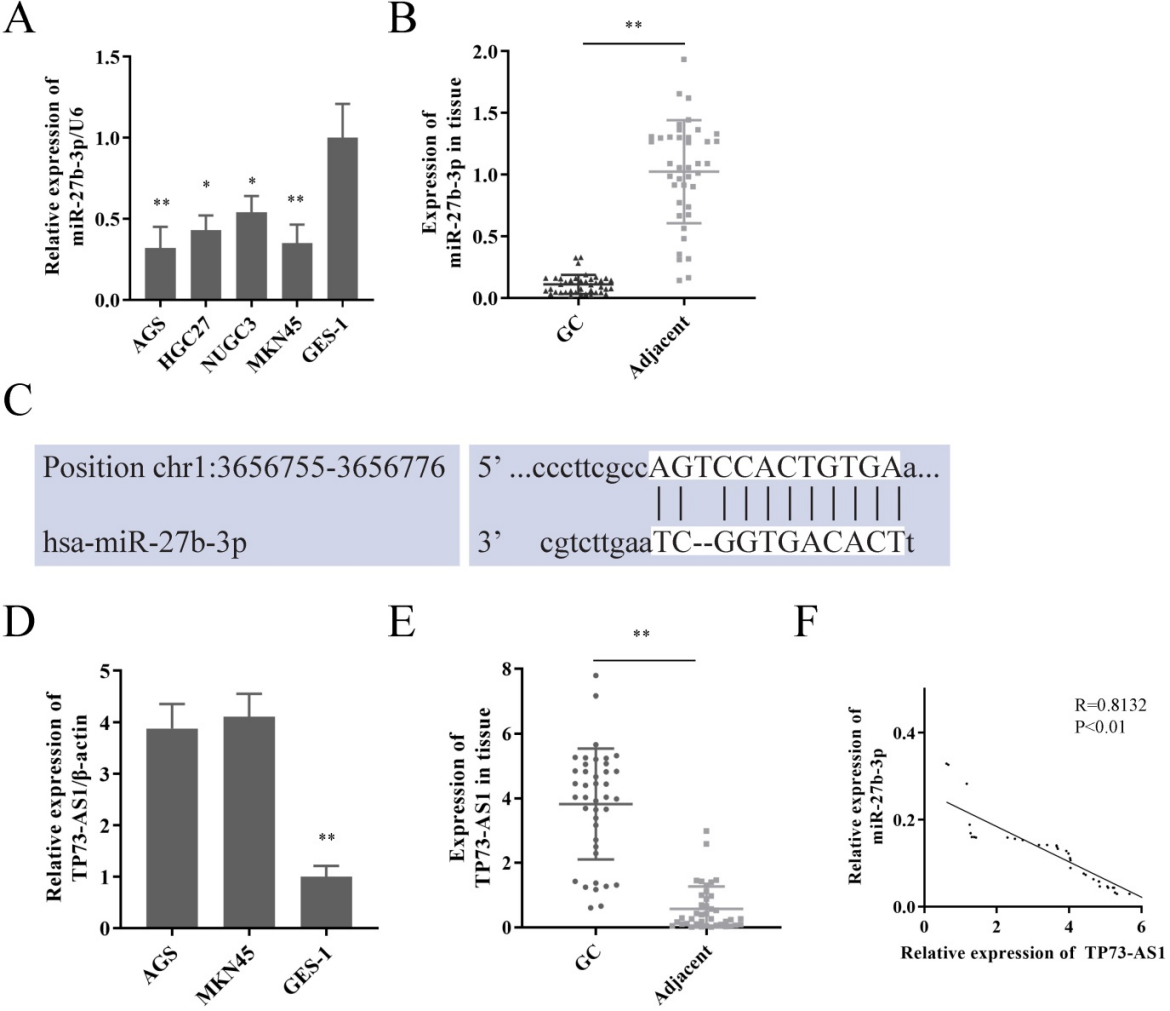
** TP73-AS1 is a target of miR-27b-3p in GC.** A. miR-27b-3p was weak expression in GC cells. B. miR-27b-3p expression in GC tissue compared to paired adjacent tissue in patients. C. The potential binding sites of TP73-AS1's 3'-UTR and miR-27b-3p transcript. D. TP73-AS1 was overexpressed in GC cells. E. TP73-AS1 high expression in GC samples. F. The expressions of TP73-AS1 and miR-27b-3p were negatively correlated.

We first reconfirmed the expression of miR-27b-3p in GC cells and tissues. Its expression was significantly decreased in GC (Fig. 1A, 1B), which was consistent with our previous research 5. Subsequently, lncRNAs that had the potential to interact with miR-27b-3p were detected by Starbase 2.0 prediction analysis 14. A high sequence match was recognized between miR-27b-3p and TP73-AS1 3'UTR (Fig. 1C). Next, we checked the expression of TP73-AS1 in GC. Obviously, TP73-AS1 was overexpressed in GC cells and tissue (Fig. 1D, 1E). The expression of TP73-AS1 and miR-27b-3p was obviously negatively correlated in GC tissue (Fig. 1F).

**Figure 2:**
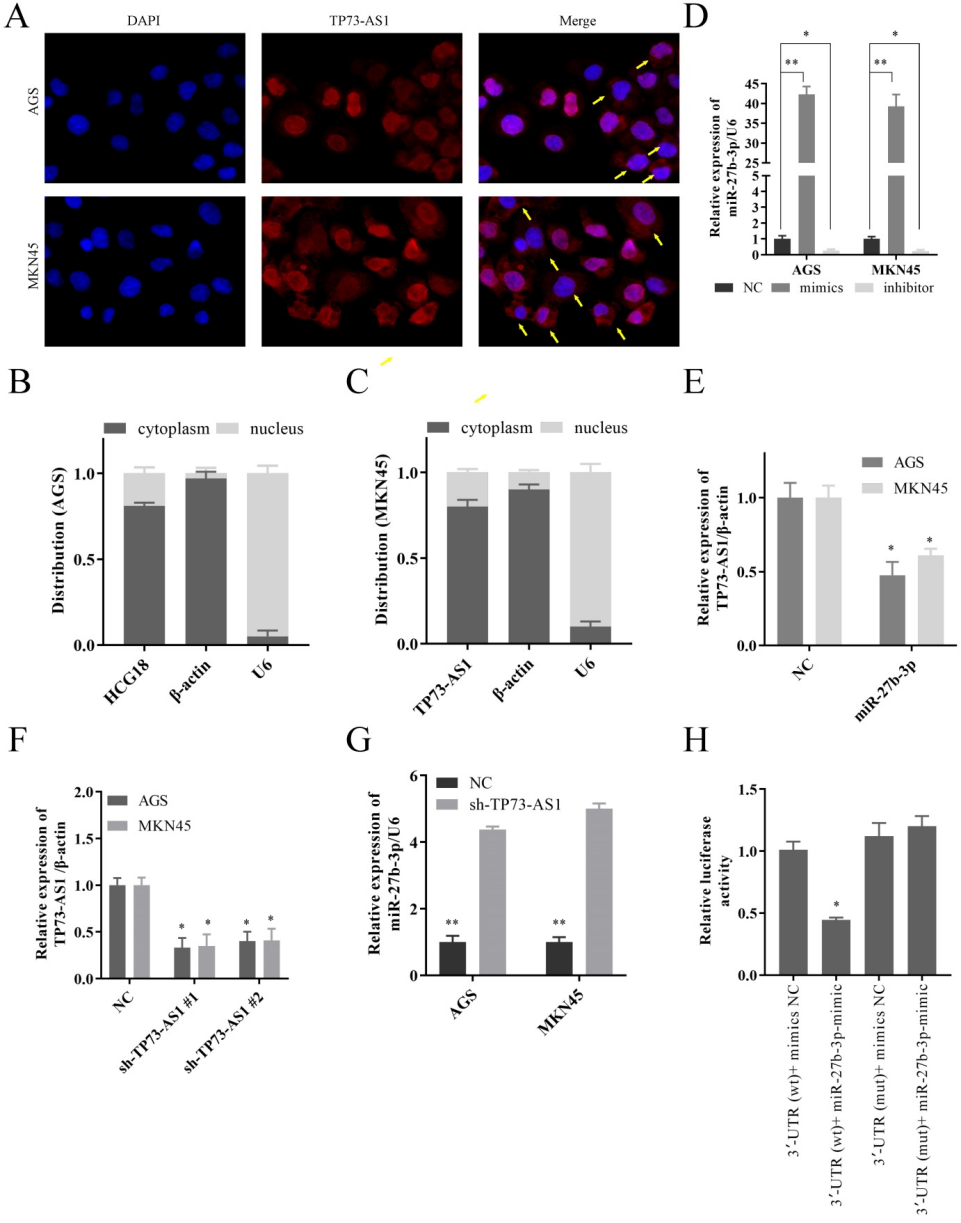
** TP73-AS1 could crosstalk with miR-27b-3p through direct binding.** A. FISH indicated that TP73-AS1 was mainly localized in the cytoplasm. B, C. RT-PCR detected the expression level of TP73-AS1 in the subcellular fractions of GC cells. U6 and β-actin were used as nuclear and cytoplasmic markers, respectively. D. The mimics and the inhibitor of miR-27b-3p could regulate its expression. E. The up-regulated miR-27b-3p inhibited the expression of TP73-AS1. F. RT-PCR analysis verifying the knockdown efficiency of shRNAs. G. The inhibition of TP73-AS1 promoted the expression of miR-27b-3p. H. Luciferase activities were measured in GC cells co-transfected with luciferase reporter containing TP73-AS1 and the mimics of miR-27b-3p.

As we all know, the subcellular location of lncRNAs determines its biological function. So, we first confirmed that TP73-AS1 was mainly overexpressed in GC cytoplasm by FISH (Fig. 2A). The Nuclear and cytoplasmic separation assay also proved this result (Fig. 2B, 2C). These suggested that TP73-AS1 mainly regulated the functions of other genes at the post-transcriptional level. The mimics and the inhibitor of miR-27b-3p could regulate its expression (Fig. 2D). After up-regulated the expression of miR-27b-3p, the expression of TP73-AS1 decreased (Fig. 2E). Similarly, down-regulated the expression of TP73-AS1 (Fig. 2F), miR-27b-3p was increased (Fig. 2G). These all further illustrated the negative regulation relationship between TP73-AS1 and miR-27b-3p. Subsequently, we constructed pGL3 luciferase reporter plasmids, which contained TP73-AS1 3'UTR wildtype sequences (TP73-AS1-wt) or mutant sequences (TP73-AS1-mut). The dual-luciferase reporter assay showed that miR-27b-3p decreased luciferase intensity of TP73-AS1-wt in HGC27-cells, while TP73-AS1-mut was unaffected (Fig. 2H).

### TP73-AS1 functioned as ceRNA of TMED5 via sponging miR-27b-3p

**Figure 3:**
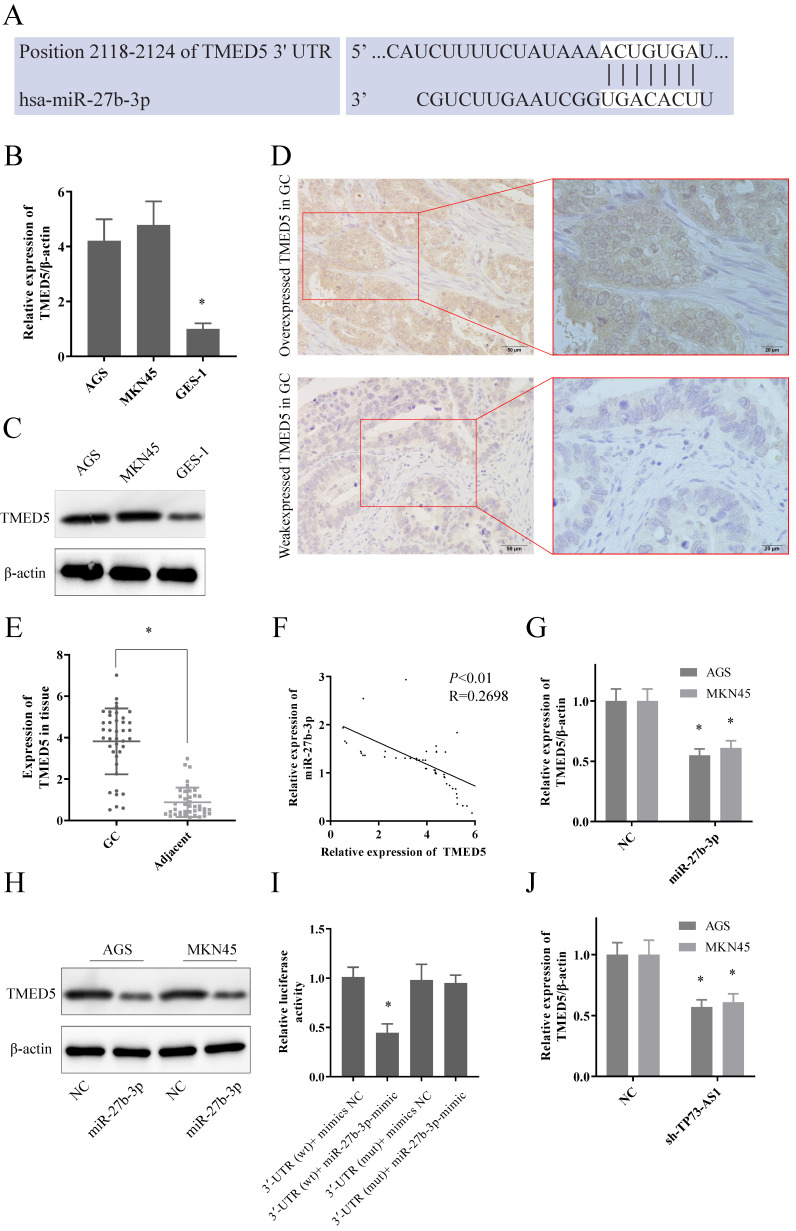
** TP73-AS1 functioned as ceRNA of TMED5 via sponging miR-27b-3p.** A. The potential binding sites of TMED5 3'-UTR and miR-27b-3p transcript. B, C. TMED5 was over-expressed in GC cells at both mRNA and protein level. D. The representative image of overexpressed TMED5 in GC (20x, 40x). E. TMED5 was over-expressed in GC tissue. F. The expressions of TMED5 and miR-27b-3p were negatively correlated. G, H. GC cells were transfected with the mimics of miR-27b-3p reduced TMED5 expression was shown by RT-RCR and western-blot. I. Luciferase activities were measured in GC cells co-transfected with luciferase reporter containing TMED5 and the mimics of miR-27b-3p. J. The expression of TMED5 was decreased with the knocked down of TP73-AS1.

We proceeded to predict the downstream target genes of TP73-AS1/miR-27b-3p. TargetScan, picTar, PITA, miRanda indicated that TMED5 might be a potential target of miR-27b-3p. Figure 3A showed the potential bind site of miR-27b-3p and 3'UTR of TMED5. Moreover, the expression of TMED5 was significantly elevated in GC both at transcriptional and protein levels (Fig. 3B, 3C). The IHC representative figure exhibited the expression of TMED5 in tumor tissues (Fig. 3D; Left: 20×; Right: 40×). The RT-PCR also showed that TMED5 was overexpressed in tumor tissues compared to adjacent (Fig. 3E). As expected, miR-27b-3p and TMED5 were also negatively correlated in GC tissues (Fig. 3F). As in tissues, the up-regulated miR-27b-3p could significantly inhibit the expression of TMED5 in both GC cells at transcript and protein level (Fig. 3G, 3H). Dual-luciferase reporter assays showed that up-regulated miR-27b-3p significantly decreased the luciferase of TMED5-wt, but the fluorescence signal of TMED5-mut was not affected (Fig. 3I). It was worth emphasizing that the inhibition of TP73-AS1 could limit the expression of TMED5 (Fig. 3K). It suggested that with the decreased TP73-AS1, miR-27b-3p bound to TP73-AS1 were released, thereby suppressing TMED5 expression. It further demonstrated the competitive binding interaction between TP73-AS1 and TMED5.

### The biological function of TP73-AS1/miR-27b-3p/TMED5 axis

**Figure 4:**
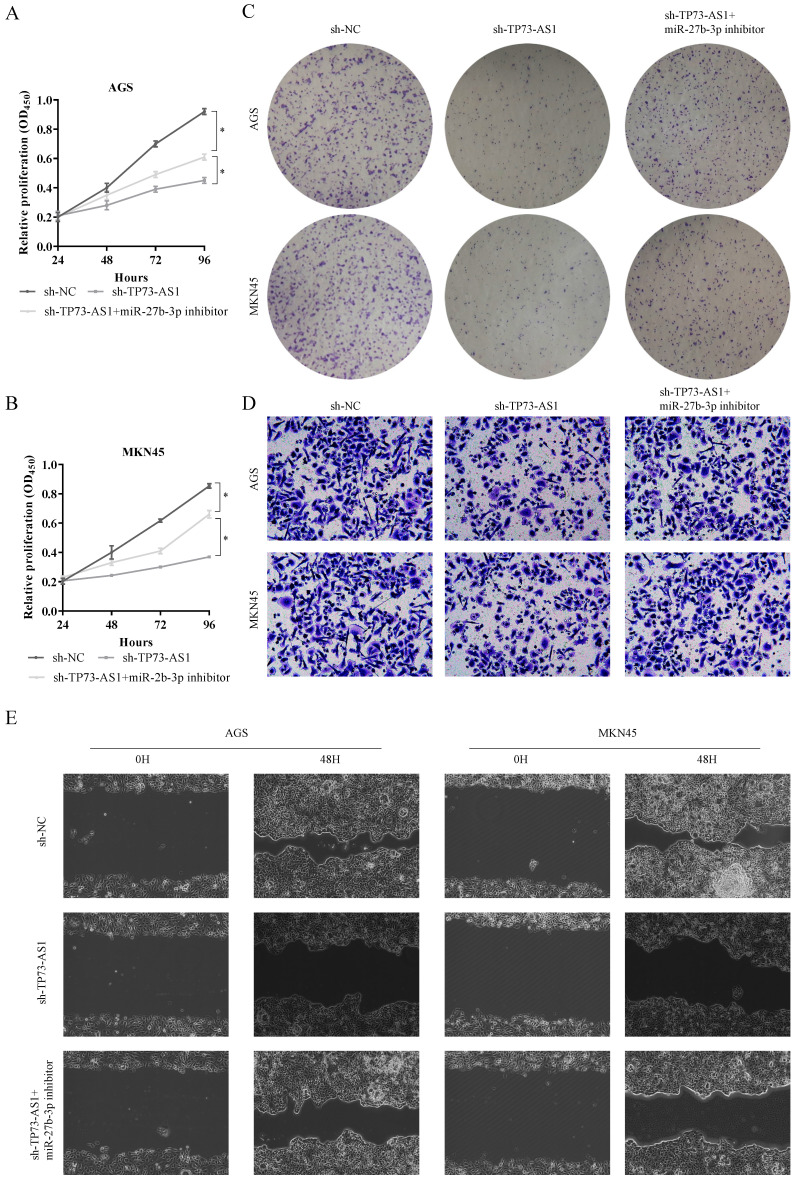
** TP73-AS1 reinforces the proliferative and invasive.** A, B. Cell proliferation assessed in TP73-AS1 knockdown and TP73-AS1 knockdown + miR-27b-3p inhibitor by CCK-8. A: AGS, B: MKN45. C. Cell proliferation assessed in TP73-AS1 knockdown and TP73-AS1 knockdown + miR-27b-3p inhibitor by colony formation. up: AGS; down: MKN45. D. Transwell assays were used to evaluate the involvement of TP73-AS1 for invasion in TP73-AS1 knockdown and TP73-AS1 knockdown + miR-27b-3p inhibitor. up: AGS; down: MKN45. E. The scrape assay confirmed that sh-TP73-AS1 could inhibit the migration of GC cells, but the miR-27b-3p inhibitor could rescue it. Data are shown as mean ± SD, n = 3. The data statistical significance is assessed by Student's* t*-test. *P < 0.05.

Furthermore, we must explore the function of the TP73-AS1/miR-27b-3p/TMED5 axis in GC. First, we used CCK-8 to evaluate the effects of TP73-AS1 and miR-27b-3p on GC cell proliferation. It indicated that the knocked down of TP73-AS1 restricted cell growth rate (Fig. 4A, 4B). On the contrary, the cell proliferation ability was partially rescued with inhibited the expression of TP73-AS1 and miR-27b-3p simultaneously (Fig. 4A, 4B). It suggested that the effect of TP73-AS1 on GC proliferation was achieved through miR-27b-3p. Colony formation was performed to verify this conclusion. The micrograph also showed that shRNA of TP73-AS1 could limit cell proliferation, and the miR-27b-3p inhibitor could antagonize this effect (Fig. 4C, 4D).

Subsequently, we also evaluated the functions of miR-27b-3p and TMED5. Obviously, inhibiting TMED5 could limit the proliferation of GC (Fig. 4E, 4F). Furthermore, miR-27b-3p inhibitor counteracted this effect (Fig. 4E, 4F). The colony formation also confirmed that the influence of TMED5 on GC was achieved through miR-27b-3p (Fig. 4G, 4H). The results confirmed the effect of TP73-AS1/miR-27b-3p/TMED5 axis on GC proliferation.

**Figure 5:**
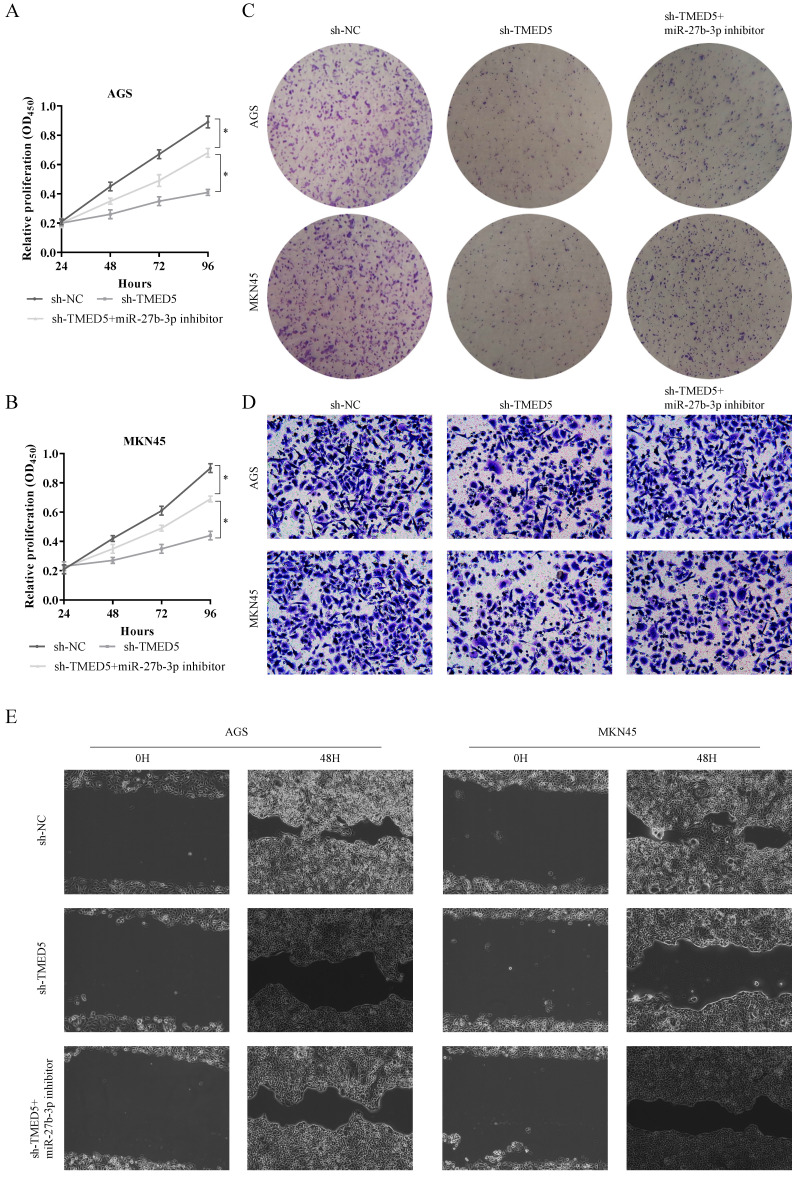
** The biological function of TP73-AS1/miR-27b-3p/TMED5 axis.** A, B. Cell proliferation assessed in TMED5 knockdown and TMED5 knockdown + miR-27b-3p inhibitor by CCK-8. A: AGS; B: MKN45. C. Cell proliferation assessed in TMED5 knockdown and TMED5 knockdown + miR-27b-3p inhibitor by colony formation. up: AGS; down: MKN45. D. Transwell assay were used to evaluate the involvement of TMED5 for invasion in TMED5 knockdown and TMED5 knockdown + miR-27b-3p inhibitor. up: AGS; down: MKN45. E. The scrape assay confirmed sh-TMED5 could inhibit the migration of GC cells, but the miR-27b-3p inhibitor could rescue it. Data are shown as mean ± SD, n = 3. The data statistical significance is assessed by Student's* t*-test. *P < 0.05.

We continued to use Transwell and scrape assay to verify the role of the TP73-AS1/miR-27b-3p/TMED5 axis in GC invasion. The Tranwell assay showed that the number of penetrating cells decreased significantly after inhibiting the expression of TP73-AS1. On this basis, when miR-27b-3p inhibitors were added, the invasive cells re-increased (Fig. 5A, 5B). Similarly, the scrape assay proved that it inhibited the expression of TMED5 and shortened the cell migration distance. Simultaneously inhibited the migration distance of miR-27b-3p and TP73-AS1cells was basically the same as the control group (Fig. 5C, 5D). The influence of TP73-AS1 on GC invasion was achieved through miR-27b-3p. Furthermore, TMED5 shRNA could also limit the number of cells passing through the membrane and inhibit cell migration (Fig. 5E-5H). The miR-27b-3p inhibitor could rescue the impact of the down-regulation of TMED5 (Fig. 5E-5H).

### TP73-AS1/miR-27b-3p/TMED5 axis regulated the wnt/β-catenin pathway

**Figure 6:**
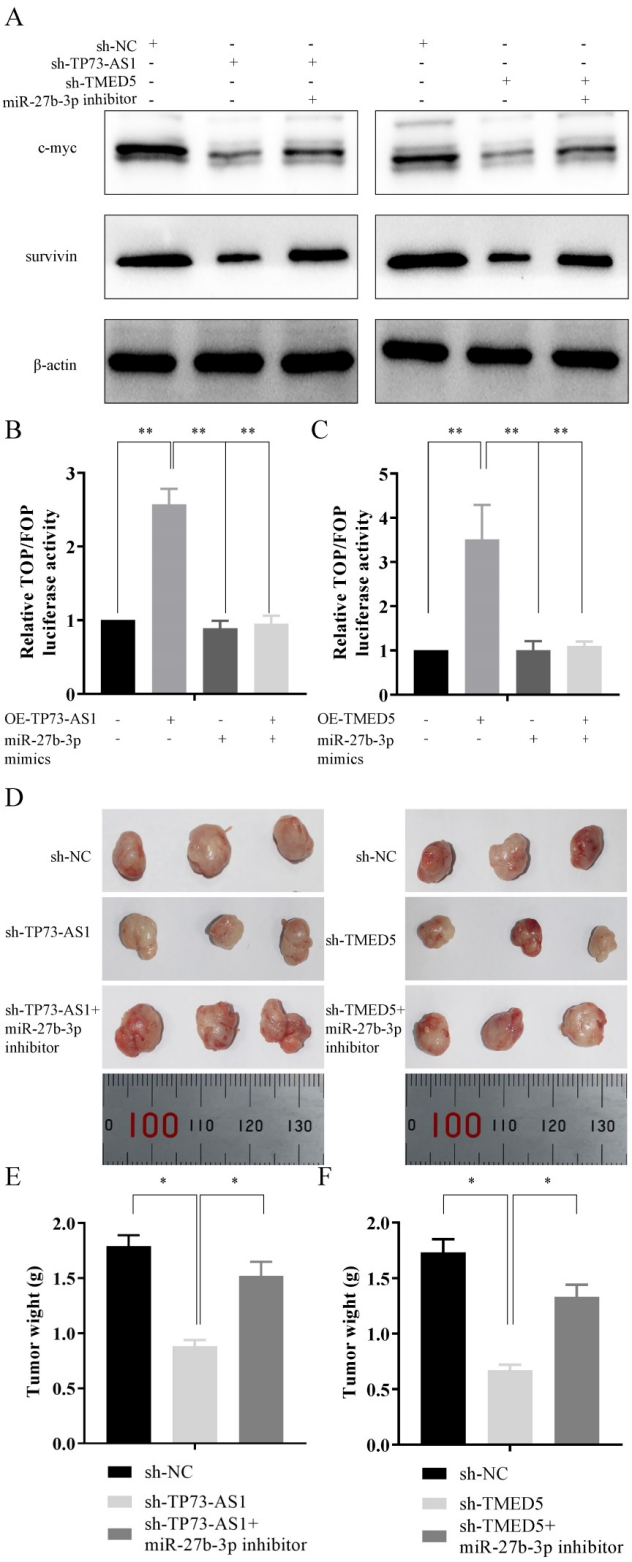
*In vivo* tumor lumps of xenograft mouse models composed of TP73-AS1, miR-27b-3p, and TMED5. A, B, C. Western blot analysis and TOP/FOP Flash reporter assays confirmed that TP73-AS1/miR-27b-3p/TMED5 axis affected wnt/β-catenin pathway. D. Representative images of the tumor lumps of each group at the endpoint of the experiment. E, F. The mean tumor weight of each group. Data are mean ± SD. of the tumor volumes, n = 5, *P < 0.05.

It had been mentioned in the literature that TMED5 might be involved in the activation of the wnt/β-catenin pathway. Thus, we explored the role of the TP73-AS1/miR-27b-3p/TMED5 axis in the wnt/β-catenin pathway. The expression of pathway essential proteins c-myc and survivin decreased after TP73-AS1 was inhibited, while their expression partially recovered after inhibition of miR-27b-3p expression (Fig. 6A). Similarly, knockdown TMED5 could also limit the expression of c-myc and survivin, while miR-27b-3p inhibitor also antagonized the function of sh-TP73-AS1 (Fig. 6A). The TOP/FOP Flash reporter assays were applied to confirm the result. We constructed the TOPFlash and FOPFlash reporters, containing wt- or mut-TCF-4 (T cell transcription factor 4) consensus binding sites to verify whether TP73-AS1/miR-27b-3p/TMED5 axis modulated the canonical wnt/β-catenin pathway. Overexpression of TP73-AS1 in AGS, the transcriptional activity of TOP/FOP was significantly enhanced, and the mimics of miR-27b-3p reversed the increasing phenomenon caused by OE-TP73-AS1 (Fig. 6B). Similar results were observed in miR-27b-3p and TMED5. Up-regulated TMED5 enhanced the transcriptional activity of TOP/FOP, while the increasing miR-27b-3p suppressed it (Fig. 6C).

### The biological function of TP73-AS1/miR-27b-3p/TMED5 axis *in vivo*

After clarified the role of the TP73-AS1/miR-27b-3p/TMED5 axis in GC *in vitro*, we needed to continue to explore its function* in vivo*. AGS cells with different treatments were injected subcutaneously into nude mice. The experiment consisted of five groups: I. AGS transfected with sh-NC; II. AGS transfected with sh-TP73-AS1 cells; III. Sh-TP73-AS1 + miR-27b-3p inhibitor transfected AGS cells; IV. AGS transfected with sh -TMED5; V. sh-TMED5 + miR-27b-3p inhibitor transfected AGS cells; (n = 3 per group). The mice were sacrificed at the end of the experiment, and the tumor weight of each group was measured (Fig. 6D-6F). The mean tumor weight at the time of death in mice injected with sh-TP73-AS1 cells was 0.88 ± 0.06 g (mean value ± SD), and the mean tumor weight of mice injected with sh-NC cells was 1.79 ± 0.10 g. Moreover, the inhibitor of miR-27b-3p could reverse it. The volume of sh-TP73-AS1 + miR-27b-3p group of tumors was 1.52 ± 0.13 g (Fig. 6D, 6E). We got similar results in TMED5 and miR-27b-3p: sh-TMED5 decreased the weight of tumors (0.62 ± 0.06 g, *vs* 1.73 ± 0.09 g, Fig. 6D, 6E). The miR-27b-3p inhibitor could rescue the weight of tumors (1.35 ± 0.10 g, Fig. 6D, 6F).

## Discussion

The formation of GC is a dynamic process, including multiple gene activation, multi-step and multi-stage completion. In different stages of its development, different components and genes participate in it. It is very beneficial for us to study gastric cancer better by studying various genes, mechanisms, and components involved in the development of GC 15, 16. In recent years, many studies have confirmed that miRNA almost participates in all cell biological processes, such as cell development, cell proliferation and apoptosis, cell migration, and invasion 17, 18. In addition, the abnormal expression of miRNA is closely related to a variety of tumors 19, 20. Moreover, some of them were thought to be related to GC development 21, 22.

Current studies have shown that MiR-27b-3p can be involved in tumor progression and metastasis through multiple pathways 23, 24. However, its expression, function, and effect on tumor prognosis are still not clarified, and there are conflicting results. For example, one report indicated that miR-27b-3p was an oncogene of breast cancer. Once miR-27b was up-regulated in breast cancer, it would cause the down-regulation of Nischarin and the activation of the NFκB signaling pathway. Finally it positive feedback promoted the increase of miR-27b transcription 25. However, another study reported that miR-27b inhibited proliferation, colony formation of breast cancer cells, increased chemosensitivity to paclitaxel, and induced cell apoptosis 26. These results indicated that miR-27b-3p might have different cellular functions in different cells. So, it is necessary to analyze further the expression regulation and up- and down-stream molecular functions of miR-27b-3p comprehensively.

We had initially clarified the anti-tumor role of miR-27b-3p in GC in our previous study 5. Therefore, our main objective in the present study was to identify the upstream molecules that could affect miR-27b-3p expression. With the development of transcriptomics, it is well established that there are numerous miRNA binding sites on various RNA transcription products 27. So, it is natural to assume that RNA transcripts containing miRNA binding sites can communicate and regulate each other by competing for shared miRNAs, thus acting as ceRNAs 28. Thus, the ceRNA mechanism confers additional non-protein-coding functions to protein-coding mRNAs. Although it has been proposed that ceRNAs may crosstalk only a tiny fraction of transcripts due to the miRNA abundance and subcellular localization 29, 30, multiple independent groups have demonstrated the regulatory function of ceRNAs in different species, including viruses, plants, mice, and humans 31. Indeed, ceRNAs have come to represent a broad network of gene regulation.

In the present study, we found that TP73-AS1 could competitively bind miR-27b-3p and thus regulated TMED5 expression. The three synergistically affected the EMT and invasion of GC. As an onco-lncRNA, TP73-AS1 could promote the malignant phenotype through multiple methods: it could maintain stemness and promote temozolomide resistance of glioblastoma multiform by affecting the expression of metabolism-related genes, ALDH1A1 32. It could also promote invasive and metastasis of ovarian cancer by regulating the expression of matrix metallopeptidases. On the other hand, the research on the mechanism of TMED5 still lacks in cancers. One research reported that the homolog of TMED5, a highly conserved type 1a transmembrane protein of the conserved g-subfamily of p24 proteins, could activate the wnt/β-catenin pathway directly in* Drosophila* 33. In addition, a study had also shown that TMED5 could interact with WNT7B in HeLa cells to activate the wnt/β-catenin pathway in cervical cancer 34. So, it is the first time that we have confirmed the activation effect of TMED5 on the wnt/β-catenin pathway in GC, thereby promoting the development and deterioration of GC.

In summary, miR-27b-3p may be a critical factor in regulating GC proliferation and invasion. The underlying mechanism is that miR-27b-3p, a competitive endogenous RNA, regulates the wnt/β-catenin pathway through the HTP73-AS1/miR-27b-3p/TMED5 axis, further affecting the malignant phenotype. These results provide new ideas for the mechanism between miR-27b-3p and GC.

## Figures and Tables

**Table 1:** TP73-AS1 expressions and clinicopathologic characteristics

Characteristics	TP73-AS1		*P*	X^2^
	High	Low		
	23	17		
**Age**			0.29	1.13
≥60	11	11		
<60	12	6		
**Gender**			0.39	0.74
Male	20	13		
female	3	4		
**Bomann tye**			0.24	2.88
I	0	2		
II	7	5		
III	16	10		
**Histology**			0.03	4.68
Adenocarcinoma	15	16		
Mixed carcinoma	8	1		
**Tumor Size**			0.80	0.06
≥5m	9	6		
<5m	14	11		
**Location**			0.82	0.40
up	5	4		
middle	6	3		
low	12	10		
**Lauren type**			0.63	0.94
Intestinal	10	10		
Mixed carcinoma	7	4		
Diffuse	6	3		
**Tumor differentiation**			0.34	0.90
Moderate and high	5	6		
Poor	18	11		
**TNM stage**			0.04	6.10
I	0	4		
II	6	4		
III	17	9		

## References

[1] Siegel RL, Miller KD, Jemal A (2020). Cancer statistics, 2020. CA: a cancer journal for clinicians.

[2] Dinmohamed AG, Visser O, Verhoeven RHA, Louwman MWJ, van Nederveen FH, Willems SM (2020). Fewer cancer diagnoses during the COVID-19 epidemic in the Netherlands. Lancet Oncol.

[3] Erratum (2020). Global cancer statistics 2018: GLOBOCAN estimates of incidence and mortality worldwide for 36 cancers in 185 countries. CA: a cancer journal for clinicians.

[4] Wei L, Sun J, Zhang N, Zheng Y, Wang X, Lv L (2020). Noncoding RNAs in gastric cancer: implications for drug resistance. Mol Cancer.

[5] Bao CH, Guo L (2021). miR-27b-3p Inhibits Invasion, Migration and Epithelial-mesenchymal Transition in Gastric Cancer by Targeting RUNX1 and Activation of the Hippo Signaling Pathway. Anticancer Agents Med Chem.

[6] Feng Q, Wu X, Li F, Ning B, Lu X, Zhang Y (2017). miR-27b inhibits gastric cancer metastasis by targeting NR2F2. Protein Cell.

[7] Tao J, Zhi X, Zhang X, Fu M, Huang H, Fan Y (2015). miR-27b-3p suppresses cell proliferation through targeting receptor tyrosine kinase like orphan receptor 1 in gastric cancer. Journal of experimental & clinical cancer research: CR.

[8] Wang XW, Liu CX, Chen LL, Zhang QC (2021). RNA structure probing uncovers RNA structure-dependent biological functions. Nat Chem Biol.

[9] He W, Liang B, Wang C, Li S, Zhao Y, Huang Q (2019). MSC-regulated lncRNA MACC1-AS1 promotes stemness and chemoresistance through fatty acid oxidation in gastric cancer. Oncogene.

[10] Guo X, Lv X, Ru Y, Zhou F, Wang N, Xi H (2020). Circulating Exosomal Gastric Cancer-Associated Long Noncoding RNA1 as a Biomarker for Early Detection and Monitoring Progression of Gastric Cancer: A Multiphase Study. JAMA Surg.

[11] Zhao J, Wu J, Qin Y, Zhang W, Huang G, Qin L (2020). LncRNA PVT1 induces aggressive vasculogenic mimicry formation through activating the STAT3/Slug axis and epithelial-to-mesenchymal transition in gastric cancer. Cell Oncol (Dordr).

[12] Zhang Q, Dou W, Taning CNT, Smagghe G, Wang JJ (2021). Regulatory roles of microRNAs in insect pests: prospective targets for insect pest control. Curr Opin Biotechnol.

[13] Salmena L, Poliseno L, Tay Y, Kats L, Pandolfi PP (2011). A ceRNA hypothesis: the Rosetta Stone of a hidden RNA language?. Cell.

[14] Li JH, Liu S, Zhou H, Qu LH, Yang JH (2014). starBase v2.0: decoding miRNA-ceRNA, miRNA-ncRNA and protein-RNA interaction networks from large-scale CLIP-Seq data. Nucleic acids research.

[15] Hutchings C, Phillips JA, Djamgoz MBA (2020). Nerve input to tumours: Pathophysiological consequences of a dynamic relationship. Biochim Biophys Acta Rev Cancer.

[16] Zhang C, Chen Z, Chong X, Chen Y, Wang Z, Yu R (2020). Clinical implications of plasma ctDNA features and dynamics in gastric cancer treated with HER2-targeted therapies. Clin Transl Med.

[17] Khan AQ, Ahmed EI, Elareer NR, Junejo K, Steinhoff M, Uddin S (2019). Role of miRNA-Regulated Cancer Stem Cells in the Pathogenesis of Human Malignancies. Cells.

[18] Sun Z, Shi K, Yang S, Liu J, Zhou Q, Wang G (2018). Effect of exosomal miRNA on cancer biology and clinical applications. Mol Cancer.

[19] Iqbal MA, Arora S, Prakasam G, Calin GA, Syed MA (2019). MicroRNA in lung cancer: role, mechanisms, pathways and therapeutic relevance. Mol Aspects Med.

[20] Shao T, Wang G, Chen H, Xie Y, Jin X, Bai J (2019). Survey of miRNA-miRNA cooperative regulation principles across cancer types. Briefings in bioinformatics.

[21] Wang R, Sun Y, Yu W, Yan Y, Qiao M, Jiang R (2019). Downregulation of miRNA-214 in cancer-associated fibroblasts contributes to migration and invasion of gastric cancer cells through targeting FGF9 and inducing EMT. Journal of experimental & clinical cancer research: CR.

[22] Xu G, Zhang B, Ye J, Cao S, Shi J, Zhao Y (2019). Exosomal miRNA-139 in cancer-associated fibroblasts inhibits gastric cancer progression by repressing MMP11 expression. Int J Biol Sci.

[23] Ye J, Wu X, Wu D, Wu P, Ni C, Zhang Z (2013). miRNA-27b targets vascular endothelial growth factor C to inhibit tumor progression and angiogenesis in colorectal cancer. PloS one.

[24] Che F, Wan C, Dai J, Chen J (2019). Increased expression of miR-27 predicts poor prognosis and promotes tumorigenesis in human multiple myeloma. Bioscience reports.

[25] Jin L, Wessely O, Marcusson EG, Ivan C, Calin GA, Alahari SK (2013). Prooncogenic factors miR-23b and miR-27b are regulated by Her2/Neu, EGF, and TNF-α in breast cancer. Cancer research.

[26] Chen D, Si W, Shen J, Du C, Lou W, Bao C (2018). miR-27b-3p inhibits proliferation and potentially reverses multi-chemoresistance by targeting CBLB/GRB2 in breast cancer cells. Cell death & disease.

[27] Ho-Xuan H, Glažar P, Latini C, Heizler K, Haase J, Hett R (2020). Comprehensive analysis of translation from overexpressed circular RNAs reveals pervasive translation from linear transcripts. Nucleic acids research.

[28] Karreth FA, Pandolfi PP (2013). ceRNA cross-talk in cancer: when ce-bling rivalries go awry. Cancer Discov.

[29] Ebert MS, Sharp PA (2010). Emerging roles for natural microRNA sponges. Current Biology.

[30] Wee LM, Flores-Jasso CF, Salomon WE, Zamore PD (2012). Argonaute divides its RNA guide into domains with distinct functions and RNA-binding properties. Cell.

[31] Tay Y, Rinn J, Pandolfi PP (2014). The multilayered complexity of ceRNA crosstalk and competition. Nature.

[32] Mazor G, Levin L, Picard D, Ahmadov U, Carén H, Borkhardt A (2019). The lncRNA TP73-AS1 is linked to aggressiveness in glioblastoma and promotes temozolomide resistance in glioblastoma cancer stem cells. Cell death & disease.

[33] Buechling T, Chaudhary V, Spirohn K, Weiss M, Boutros M (2011). p24 proteins are required for secretion of Wnt ligands. EMBO reports.

[34] Yang Z, Sun Q, Guo J, Wang S, Song G, Liu W (2019). GRSF1-mediated MIR-G-1 promotes malignant behavior and nuclear autophagy by directly upregulating TMED5 and LMNB1 in cervical cancer cells. Autophagy.

